# Hemi-capsulo-rhombencephalic demyelination

**DOI:** 10.4103/0972-2327.64631

**Published:** 2010

**Authors:** C. J. Suresh Chandran, V. Maheshwaran, Madhavan Unni

**Affiliations:** Department of Neurology, Kerala Institute of Medical Sciences, Trivandrum, Kerala, India; 1 Department of Radiology, Kerala Institute of Medical Sciences, Trivandrum, Kerala, India

A 30-year-old male presented with acute-onset headache, vomiting, and facial deviation to the left. He had had an upper respiratory infection 1 week earlier that had subsided within 2 days. Clinical examination showed right gaze palsy, right lower motor neuron (LMN) facial palsy, and minimal right cerebellar signs. Fundus examination was normal and meningeal signs were absent.

**Figure 1:**
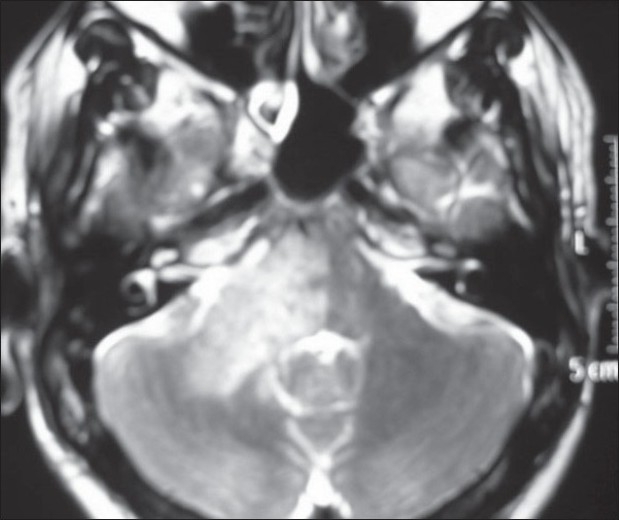
Axial T2 image showing hyperintensity in the right pons and cerebellum

**Figure 2:**
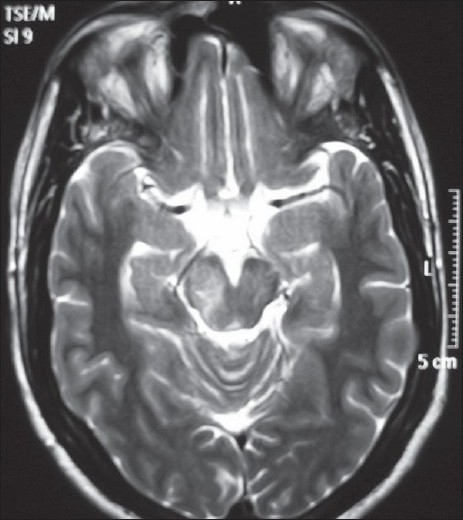
Axial T2 image showing hyperintensity in the right half of the midbrain. Note that the right side of the midbrain is swollen

**Figure 3:**
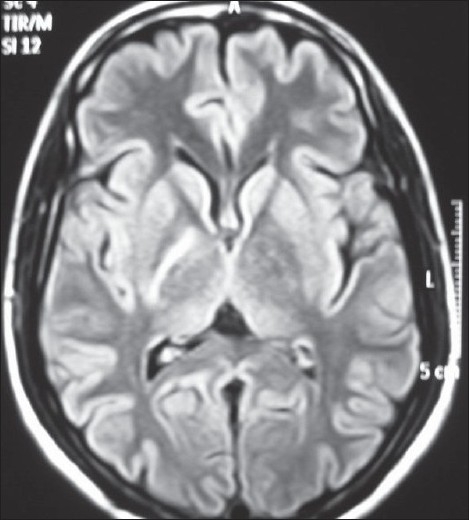
Axial FLAIR image showing hyperintensity of the posterior limb of the right internal capsule

**Figure 4:**
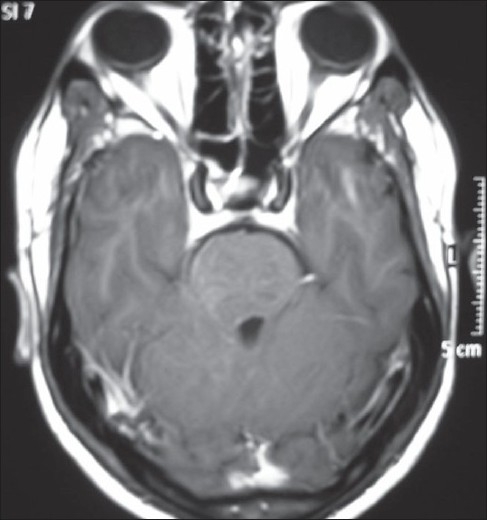
Axial T1 contrast image showing a swollen right half of pons. The lesions are isointense on T1, without any enhancement. Note the compression of the fourth ventricle from the right side

MRI brain showed hyperintense lesions in T2 and FLAIR images, involving the right half of the brainstem, the right cerebellum, and the posterior limb of the right internal capsule. These lesions were isointense on T1 and did not show any diffusion restriction. The right half of the brainstem was swollen. No significant contrast enhancement was noted [Figures 1‐4].

**Figure 5:**
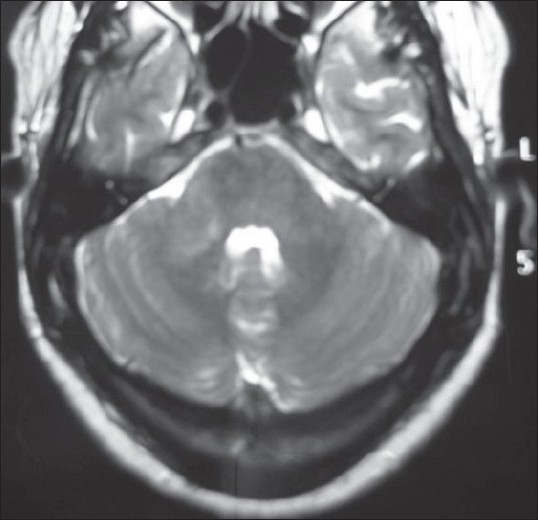
MRI (T2 axial) done 4 weeks after presentation showing resolution of the lesions

MR angiography and MRI spine were normal. Cerebrospinal fluid (CSF) study showed elevated protein (90 mg/dl), normal sugar, and 5 lymphocytes/mm^3^ CSF oligoclonal band, Indian ink staining, and HSV PCR were negative. Gram's stain did not yield any organisms. Vasculitic workup, HIV test, and serum and CSF VDRL were negative. We diagnosed acute disseminated encephalomyelitis (ADEM) – right hemi-capsulo-rhombencephalic demyelination. The patient was treated with intravenous methyl prednisolone 1 gm once daily for 3 days, followed by oral prednisolone 1 mg/kg for 2 weeks. The neurological deficits resolved in 1 week. Repeat MRI done 4 weeks later showed resolution of the lesions [Figure 5].

ADEM is a monophasic inflammatory demyelinating disease of the central nervous system, characterized by scattered focal or multifocal (disseminated) inflammation of the brain and/or spinal cord. A number of variants of ADEM have been described, namely, tumefactive demyelination (Marburg disease), acute hemorrhagic leukoencephalitis (AHLE), Balo's concentric sclerosis, and focal or site-restricted forms (e.g., optic neuritis, myelitis, and cerebellitis).[1] Our case was peculiar in that that demyelination was limited to the right half of the brainstem, the right cerebellum, and the right internal capsule. Although there was extensive involvement seen in MRI, the clinical deficits were not as marked and response to treatment was good. Listerial infection is a possibility worth considering in this case in view of the predominant rhombencephalic involvement;[2]however, the absence of CSF pleocytosis, the Gram's stain result, the presence of capsular involvement, and the response to steroids ruled it out.

Unilateral involvement is rare in ADEM. There have been rare reports of hemicerebellitis in children and solitary hemispheric tumefactive demyelinating lesions have been reported.[3-5] ADEM presenting as hemiplegia and ataxic hemiparesis have been reported, but MRI shows bilateral involvement in these cases.[6,7] Inflammatory disorders with unilateral brain involvement include Rasmussen's encephalitis, primary angitis of the central nervous system, and herpes zoster–related vasculopathy. Rasmussen's encephalitis is an inflammatory immune-mediated brain disorder characterized by unilateral hemispheric atrophy, intractable seizures, and progressive neurological dysfunction. MRI features of Rasmussen's encephalitis include unilateral enlargement of CSF compartments (most accentuated in the insular and periinsular regions), with increased cortical and/or subcortical T2 and FLAIR signals and caudate head atrophy.[8]In primary angitis of the central nervous system the common pattern of parenchymal involvement is multifocal, unilateral, proximal lesions in the anterior circulation.[9] Herpes zoster–related vasculopathy also shows unilateral lesions in the anterior or middle cerebral artery territory.[10]

To the best of our knowledge, no cases of unilateral demyelination involving the brainstem and internal capsule have been reported earlier. Our case of hemi-capsulo-rhombencephalic demyelination is thus a unique case of site-restricted ADEM.
